# Model of Risk of Exposure to Lyme Borreliosis and Tick-Borne Encephalitis Virus-Infected Ticks in the Border Area of the Czech Republic (South Bohemia) and Germany (Lower Bavaria and Upper Palatinate)

**DOI:** 10.3390/ijerph16071173

**Published:** 2019-04-02

**Authors:** Václav Hönig, Pavel Švec, Lukáš Marek, Tomáš Mrkvička, Zubriková Dana, Maria (Vögerl) Wittmann, Ondřej Masař, Daniela Szturcová, Daniel Růžek, Kurt Pfister, Libor Grubhoffer

**Affiliations:** 1 Institute of Parasitology, Biology Centre, Academy of Sciences of Czech Republic, Branisovska 31, 370 05 Ceske Budejovice, Czech Republic; ruzekd@paru.cas.cz (D.R.); liborex@paru.cas.cz (L.G.); 2 Faculty of Science, University of South Bohemia, Branisovska 31, 370 05 Ceske Budejovice, Czech Republic; 3 Veterinary Research Institute, Hudcova 296, 621 00 Brno, Czech Republic; 4 Department of Geoinformatics, VSB—Technical University of Ostrava, 17. listopadu 15, 708 00 Ostrava, Czech Republic; pavel.svec1@vsb.cz (P.S.); ondrej.masar@gmail.com (O.M.); daniela.szturcova@vsb.cz (D.S.); 5 GeoHealth Laboratory, Geospatial Research Institute, University of Canterbury, Private Bag 4800, Christchurch 8140, New Zealand; lukas.marek@canterbury.ac.nz; 6 Faculty of Economics, University of South Bohemia, Studentska 13, 370 05 Ceske Budejovice, Czech Republic; mrkvicka.toma@gmail.com; 7 Institute of Comparative Tropical Medicine and Parasitology, Ludwig-Maximilians-Universität München, Leopoldstr. 5, 80802 Munich, Germany; cernanska@saske.sk (Z.D.); m.voegerl@googlemail.com (M.(V.)W.); kpfister@duc.ch (K.P.); 8 Institute of Parasitology, Slovak Academy of Sciences, Hlinkova 3, 040 01 Kosice, Slovakia

**Keywords:** tick, Lyme borreliosis, tick-borne encephalitis, *Ixodes ricinus*, risk modeling, geographical information systems

## Abstract

In Europe, Lyme borreliosis (LB) and tick-borne encephalitis (TBE) are the two vector-borne diseases with the largest impact on human health. Based on data on the density of host-seeking *Ixodes ricinus* ticks and pathogen prevalence and using a variety of environmental data, we have created an acarological risk model for a region where both diseases are endemic (Czech Republic—South Bohemia and Germany—Lower Bavaria, Upper Palatinate). The data on tick density were acquired by flagging 50 sampling sites three times in a single season. Prevalence of the causative agents of LB and TBE was determined. Data on environmental variables (e.g., altitude, vegetation cover, NDVI, land surface temperature) were obtained from various sources and processed using geographical information systems. Generalized linear models were used to estimate tick density, probability of tick infection, and density of infected ticks for the whole area. A significantly higher incidence of human TBE cases was recorded in South Bohemia compared to Bavarian regions, which correlated with a lower tick density in Bavaria. However, the differences in pathogen prevalence rates were not significant. The model outputs were made available to the public in the form of risk maps, indicating the distribution of tick-borne disease risk in space.

## 1. Introduction

Vector-borne diseases represent a serious health risk to human and animals in many parts of the world. In Europe, pathogens transmitted by *Ixodes ricinus* (*I. ricinus*) ticks, namely agents of Lyme borreliosis (LB) and tick-borne encephalitis (TBE), are the most widespread vector-borne diseases of human [1,2]. To be able to take appropriate measures to limit the level of exposure of human populations to vector-borne pathogens, detailed knowledge of the distribution of the risk of infection in space and time is needed.

Lyme borreliosis is caused by spirochetes of the *Borrelia burgdorferi* sensu lato complex (*Spirochaetales*, *Spirochaetaceae*) and TBE by the tick-borne encephalitis virus (*Flavivirus*, *Flaviviridae*). Both the diseases are zoonoses maintained in geographically more or less specified areas in nature, called “natural foci”. Humans are accidental, predominantly dead-end hosts, which get infected when entering the natural focus. In these foci, causative agents of vector-borne diseases are maintained by circulation between arthropod vectors and transmission competent hosts. Therefore, the epidemiology of these infections is influenced not only by the intrinsic features of the pathogen, but also by the behavior and ecology of its vectors and hosts. Moreover, all three components of vector-borne pathogen natural cycles are influenced by numerous environmental factors of abiotic (temperature, air humidity, access of sunlight, altitude etc.) [3,4,5,6] or biotic (natural host availability, transmission competence of the host, vector-host contact rate, host immunity etc.) [7,8,9] nature. Because the environment influences all three components of the natural circulation, environmental variables may be used for the prediction of risk of infection for humans entering certain areas [10,11].

The existing models for tick-borne pathogens created for ecological or epidemiological purposes are usually based on the estimation of host-seeking tick density and/or pathogen prevalence in the field, collection of different environmental data, and use of various statistical approaches (multiple linear regression, classification and regression trees, generalized linear models etc.) in order to find a mathematical expression of the relationship between the environmental variables and characteristics of tick and pathogen distribution [6,12,13,14,15,16,17].

The main aim of this study was to use a set of tick abundance and pathogen prevalence data to prepare summarized, easy to access information on the risk of infection by tick-borne diseases for the general public. Based on the results of an extensive field sampling and laboratory analysis of the tick populations [18,19], we have developed a model for the prediction of the density of host-seeking *I. ricinus* ticks, LB spirochete and TBEV prevalence, and density of LB spirochete/TBEV-infected ticks using commonly available environmental data. Using tools of geographic information systems (GIS), we have integrated the results of the research, including the risk models, in map outputs which have been made available to the public through an interactive internet-based map portal.

## 2. Materials and Methods

### 2.1. Study Sites

**Figure 1:**
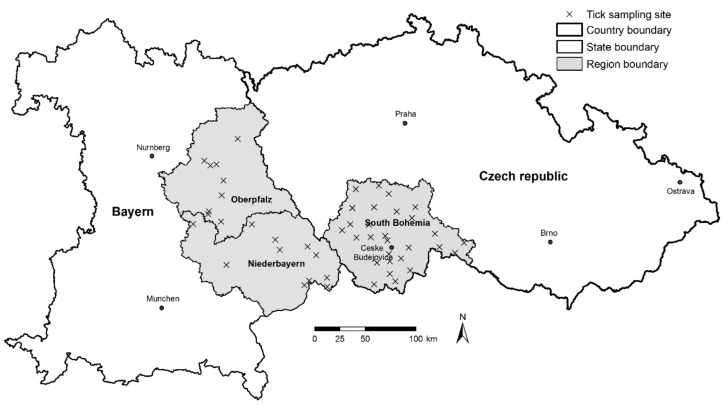
Surveyed area of South Bohemia (Czech Republic), Lower Bavaria and Upper Palatinate (Bavaria, Germany); Lower Bavaria—Niederbayern, Upper Palatinate (Oberpfalz), Bayern—Bavaria; localization of tick sampling sites indicated by a cross sign.

The data on the distribution of *I. ricinus* tick density, and TBEV and *B. burgdorferi* s.l. prevalence in ticks were collected in the Czech-German borderland in 2010–2011. Records of retrospective LB and TBE human disease case incidence were obtained for the same area for the period 2001–2009. The study area consisted of the region of South Bohemia in the Czech Republic and the regions of Lower Bavaria and Upper Palatinate of the German Free State of Bavaria (Figure 1). Geographically, climatically, and biologically, the area is considerably heterogeneous ranging from Danube Valley at the German-Austrian border to the highest sections of Šumava (Bohemian Forest) and Bavarian Forest mountain range (280–1450 m a.s.l.). The total area under survey comprised 30,077 km^2^ (10,056 km^2^ in the Czech Republic and 20,021 km^2^ in Germany). The regions of South Bohemia and Bavaria are recognized as areas of endemic occurrence of LB and TBE [20,21].

The acarological risk of tick-borne disease infection is determined by the prevalence of the pathogen in ticks and abundance of the tick population. Previous studies have shown that both the parameters may be predicted based on their known relationship to certain environmental factors [6,10,22,23]. Therefore, in the field, in a network of 50 study sites spread throughout the study area, we have collected data on the density of host-seeking *I. ricinus*, *B. burgdorferi* s.l., and TBEV prevalence. In parallel, a large dataset of environmental data was established and subsequently used to generate predictions of host-seeking tick density and pathogen prevalence for every pixel of the study area.

### 2.2. Field Study

The distribution of ticks and tick-borne pathogens was estimated in a network of 50 study sites (30 in South Bohemia, 20 in Bavaria—Lower Bavaria and Upper Palatinate) (Figure 1), as described in [18]. The density of host-seeking *I. ricinus* ticks was estimated by “flagging” each sampling site (600 m^2^ per site and sampling event) three times per year (May, June/July, September/October), resulting in 150 separate entries (for details, please see [18]). Only nymphal and adult *I. ricinus* ticks were sampled, as the density of larval ticks is difficult to estimate due to their highly aggregated distribution, they rarely infest humans [24], and they generally have a low pathogen prevalence [25].

Collected ticks were transported to the laboratory and tested for the presence of DNA of *B. burgdorferi* and TBEV RNA using conventional PCR for borrelia [26,27], reverse transcription followed by conventional PCR for TBEV (samples from South Bohemia) [28], and reverse transcription followed by real-time PCR (samples from Bavaria) [29] (for more detailed information see [18] and [19]). The data for both countries were analyzed as a single dataset. Altogether, data from 15,150 and 28,862 ticks were available considering *B. burgdorferi* s.l. and TBEV infection status, respectively.

### 2.3. Environmental Data

Apart from the data on tick and pathogen distribution, we have used various sources to collect epidemiological (numbers of disease cases), demographic (numbers of inhabitants), and environmental data (altitude, slope, exposition, vegetation cover, temperature etc.). The types of data and their respective sources are summarized in Table 1. Satellite images acquired by the MODIS instrument were used as a source of land surface temperature (LST) and vegetation cover information—normalized difference vegetation index (NDVI). From the NASA Land Processes Distributed Active Archive Center, the following products were downloaded for the whole area under survey: Surface Temperature & Emissivity 8-Day L3 Global, MOD11A2, and Vegetation Indices 16-Day L3 Global MOD13Q1. Individual measurements were extracted for the sampling localities and specified dates.

Using GIS, the data were extracted and optimized to enter the analysis. With regard to differences in the resolution of the source data, we have worked with an optimized resolution of the whole dataset of a 250 m pixel size. Minimum, maximum, mean seasonal (May–September), and mean annual LST and NDVI values were calculated for individual sampling sites. In the case of physical geographical characteristics and forest type, a buffer area around the collection site was taken into account (pixel of collection site and eight surrounding pixels). The characteristics were expressed as the proportion of the area of a given quality (e.g., covered by deciduous forests). The density of host-seeking ticks was expressed as the number of ticks per 100 m^2^, and prevalence as portion of samples positive for LB spirochetes or TBEV of total samples tested.

Epidemiological data were obtained (National Institute of Public Health, Czech Republic; Robert Koch Institute and local public health authorities, Germany) as numbers of human disease cases registered between 2001–2009 in each of the 626 municipalities in South Bohemia and 536 in the selected regions of Bavaria. Data on TBE and LB were available for South Bohemia, where both diseases are reported mandatory. In the case of Lower Bavaria and Upper Palatinate, only data on TBE could be gathered. Census of population and housing data were used as the source of numbers of inhabitants. Disease incidence was expressed as the number of cases per 100,000 inhabitants.

### 2.4. Model Construction

Independent models were created for the prediction of the density of host-seeking ticks (nymphal and adult *I. ricinus*), and probability of infection of a tick by LB spirochetes and TBEV. Subsequently, the tick density model was combined with either the LB spirochetes or TBEV infection probability model, giving a prediction of the density of infected ticks as a measure of risk of infection for an unvaccinated person entering a particular geographical area.

First, the correlation of all potential explanatory variables was examined by the Spearman ranking test, and cross-correlations among the variables were inspected. Significantly cross-correlated explanatory variables were grouped together. Subsequently, the set of potential explanatory variables was tested for correlation with the dependent variables. From each group of cross-correlated explanatory variables, one representative with the highest correlation coefficient with the dependent variable was chosen for further analysis.

Generalized linear models were used for host-seeking tick density prediction. With regard to the character of the data, a log-linear model with a Poisson distribution (Poisson regression model) was applied. Individual explanatory variables were evaluated based on a Likely-hood type 3 test. The quality of the model was evaluated using McFadden’s pseudo R^2^.

In the case of the prediction of *B. burgdorferi* s.l. and TBEV infection in ticks, a logit model was applied. The relationship of a bivariate dependent variable (tick infected/tick not infected) with potential explanatory variables of the same dataset as in the case of host-seeking tick density was examined in the model. First, all the candidate explanatory variables were included in the model and subsequently, the variables that were not statistically significantly important were excluded, as is done using a backwards stepwise approach. Individual variables were excluded based on the comparison of Wald statistics and (cross-) correlation coefficient values. BIC and AIC criteria were controlled, indicating the performance of the whole model. The performance of the two resulting infection probability models was evaluated using ROC (Receiver Operating Characteristic) analysis. An ROC curve evaluates the relationship between the sensitivity (true positive fraction) and specificity (false positive fraction) of the model [30] and it is a suitable measure for the binary classification. The common way of interpretation of the ROC is quantifying the fraction of an area under the ROC curve (AUC). Generally speaking, the higher the AUC, the better the ability of the model to classify. A meaningful model should have an AUC higher than 0.5, which indicates that it is better than a random predictor [31].

### 2.5. Statistical and GIS analyses

Statistical analyses were performed using STATISTICA ver. 9 (StatSoft CR, Prague, Czech Republic) and R [32]. A non-parametric Mann-Whitney test was used for a comparison of disease incidence rates and host-seeking tick densities, and a chi square test for a comparison of the prevalence rate (LB) or minimum infection rate (TBEV). Differences with *p* < 0.05 were considered statistically significant. All GIS analyses were performed and map outputs generated using ArcGIS 10.X (ESRI, Redlands, USA). The project outputs were integrated in a web-based map portal using ArcGIS for Server (ESRI, Redlands, USA).

## 3. Results

### 3.1. Disease Incidence

In the period of 2001–2009, altogether, 1453 TBE cases were registered in South Bohemia compared to 417 cases reported in Lower Bavaria and Upper Palatinate. When expressed as incidence, 231 TBE cases per/100,000 inhabitants were reported from the South Bohemian region compared to 18 cases from Bavaria. The differences were found to be statistically significant (Mann-Whitney *U* Test, *p* < 0.0001).

### 3.2. Density of Host-Seeking I. Ricinus Ticks and Pathogen Prevalence

All the analyses were based on aggregated data from South Bohemia and Bavaria, resulting in 50 study sites, 150 individual sampling events, 15,150 *I. ricinus* ticks analyzed for the presence of *B. burgdorferi* s.l., and 28,862 ticks tested for TBEV. The mean density of host-seeking ticks reached 30.1 nymphs/100 m^2^ (range 1.3–110.2), and 2.0 adult ticks/100 m^2^ (0–10.7). The mean density of all host-seeking ticks and nymphal ticks in South Bohemian sampling sites reached significantly higher values (37.4 ticks/100 m^2^ and 34.9 nymphs/100 m^2^) than the abundance of host-seeking ticks in Bavarian sites (24.5 ticks/100 m^2^ and 22.8 nymphs/100 m^2^) (Mann-Whitney U test, *p* < 0.001). In the case of adult ticks, the difference was not statistically significant (2.3 adult ticks/100 m^2^ in South Bohemia and 1.7 adults/100 m^2^ in Bavaria).

The total prevalence of LB spirochetes reached 12.3% (1865 positive samples/15,150 tested), and the minimum infection rate (MIR) (samples tested in pools) of TBEV reached 0.30% (87/28,862). Slightly higher prevalence rates of *B. burgdorferi* were observed in samples from Bavaria, whereas higher MIRs of TBEV were found in South Bohemian samples. The only statistically significant differences (χ^2^ test; *p* < 0.01) were found between the *Borrelia* prevalence in adult ticks, which was significantly higher in samples from Bavarian localities (Table 2 and Table 3). Ticks positive for DNA of *B. burgdorferi* s.l. were found at each of the 50 study sites. At least one TBEV positive tick was sampled at 21 of 30 (70%) localities in South Bohemia and at 7 of 20 (35%) study sites located in Bavaria.

### 3.3. Host-Seeking Tick Density Model

Based on the Spearman ranking test, a set of potential explanatory variables was identified (Table 4) to be included in the initial model. Altitude as a well-known factor influencing tick density was included in the model, despite its poor correlation with the dependent variable in the test. Furthermore, significant differences were found among the different sampling events spring—summer—autumn (ANOVA; *p* < 0.01) and thus the sampling event was added to the model as a categorical predictor.

A log-linear model with a Poisson distribution was used for host-seeking tick density prediction. According to the residual analysis, an outlier was identified and excluded from further analysis. All included variables were found to be significant in the model (Likely-hood type 3 test, *p* < 0.001). The parameters were estimated, resulting in the following equation (Table 5):

ln (*host-seeking tick density*) = 5.973866 + *season* + 0.019914 *LST Seasonal Mean* − 0.000770 *Altitude* − 0.492528 *Portion Southwards* − 1.046671 *NDVI August*

For the only categorical variable, “season”, the following parameters were estimated: spring = 0.393470, summer = 0.096751, autumn = 0. Thus, three independent models were created for the different parts of the tick season. 

**Figure 2:**
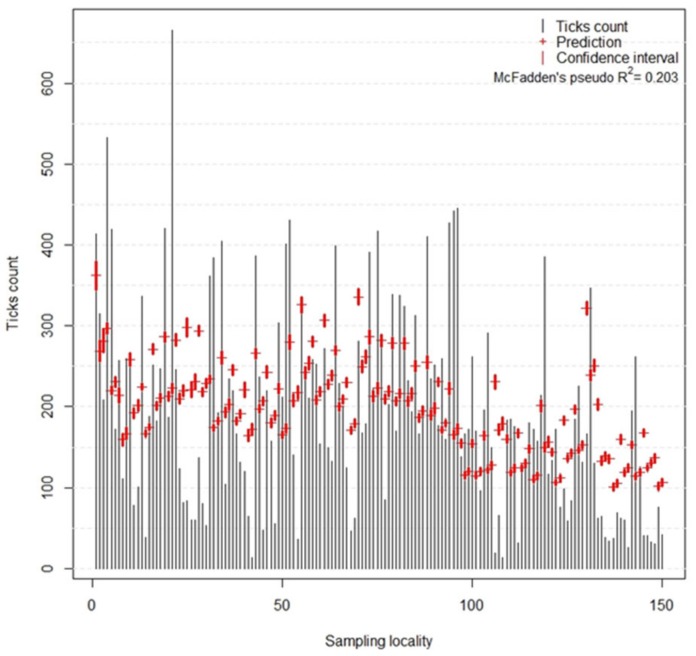
Comparison of observed (determined in field) and predicted (estimated by the model of host-seeking tick density) *I. ricinus* tick counts on individual sampling sites. Grey lines show the observed values, red crosses indicate the value predicted by the model, and red lines represent the confidence interval.

The McFadden’s pseudo R^2^ of the presented model reached the value of 0.203, indicating a model with very good fit. The comparison of observed and predicted tick counts on individual locations together with its confidence interval is depicted in Figure 2.

### 3.4. B. burgdorferi and TBEV Probability of Infection Model

The probability of infection of a tick by *B. burgdorferi* s.l. or TBEV was predicted using a logit model. The parameter estimates and goodness of fit data for the probability of infection of the tick by *B. burgdorferi* and TBEV are presented in Table 6 and Table 7, respectively. Season of collection, the only categorical explanatory variable, was not found to be significant for either *Borrelia* or for the TBEV risk model.

The probability of infection of a tick by spirochetes of Lyme borreliosis was expressed by the following equation (Φ standing for the normal distribution function):

Probability of *B. burgdorferi* infection = Φ (0.546025 − 1.064178 NDVI May − 0.153217 Portion Northwards + 0.000483 Altitude − 0.051443 LST Seasonal Mean)

The probability of infection of a tick by TBEV was expressed by the following equation (Φ standing for the normal distribution function):

Probability of TBEV infection = Φ (−2.275259–0.038585 LST seasonal minimum + 0.218823 Mixed forest portion − 2.526153 NDVI May + 2.323306 NDVI seasonal maximum).

**Figure 3:**
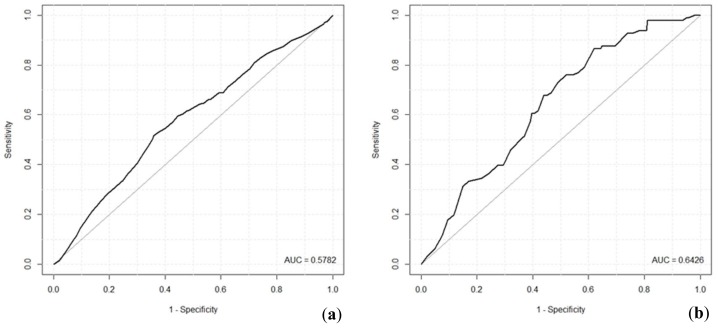
Receiver operating characteristics (ROC) of classification ability of *B. burgdorferi* infection probability model (**a**) and TBEV infection probability model (**b**); AUC—area under ROC curve.

Validation of the classification ability of the previous two models was evaluated using ROC analysis. ROC curves are depicted in Figure 3, together with their AUC. AUC of the model for *B. burgdorferi* was 0.58, while the model for TBEV infection reached the level of 0.64.

**Figure 4:**
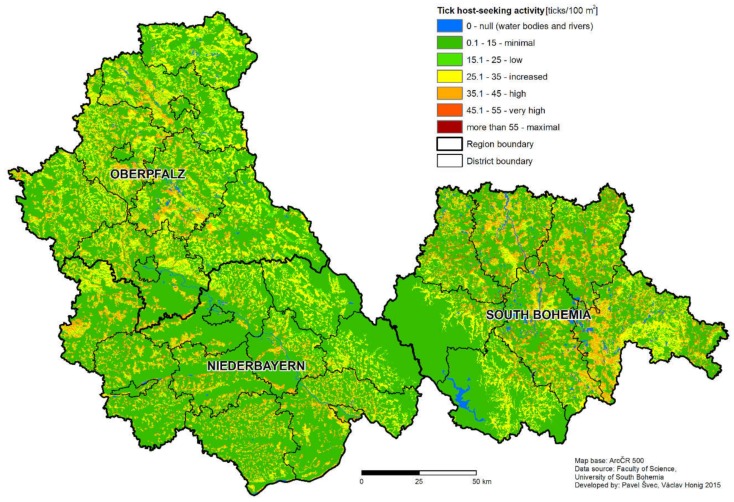
Map output of the model of the host-seeking tick density for South Bohemia (Czech Republic), Lower Bavaria and Upper Palatinate; Lower Bavaria—Niederbayern, Upper Palatinate (Oberpfalz). The level of the risk of encountering a host-seeking tick is indicated by color coding—blue—no risk, brown—maximum risk). The values of host-seeking tick density are only indicative and may change due to annual fluctuations.

By joining the model of host-seeking tick density prediction with either the model of probability of *B. burgdorferi* or TBEV infection, a risk model predicting the density of infected ticks was created. The mathematical models were integrated into the GIS system and risk maps were generated (Figure 4). 

Comparison of the classification (prediction of general density of ticks without the influence of season) of the South Bohemian and Bavarian regions is presented in Table 8. Furthermore, all the epidemiological data and risk models were made available for the general public via an online map portal (http://gisak.vsb.cz/klistata/index_en.html).

## 4. Discussion

Tick-borne diseases are recognized as a well-known health threat in the Northern Hemisphere [33]. Our study was carried out in a Czech-German cross-border area offering two regions with relatively similar environmental conditions in two neighboring countries. The epidemiological survey has shown a high difference in the occurrence of human TBE disease cases. Far higher numbers of TBE cases were reported from South Bohemia than from regions of Lower Bavaria and Upper Palatinate in Bavaria, both in absolute numbers as well as in relative numbers per 100,000 inhabitants or km^2^. The possible reasons for this may lie in a higher abundance of tick populations, higher prevalence of TBEV, or higher activity of infection-susceptible humans within the TBEV foci. Differences in the case recognition and case reporting do not seem very likely, because of the well-established case definition and reporting system based on the localization of tick attack (instead of place of patient´s residence). Moreover, the region of South Bohemia has in the long term, by far the highest incidence of TBE among the regions of the Czech Republic, which share the same reporting system [20]. Additionally, there is no striking difference in the vaccination rate [34,35] between the two areas. One of the previously described specific factors, which in fact increases human activity in South Bohemia, is its high popularity as a place for weekend/vacation leisure activities (mostly associated with spending time outdoors) [36,37]. Due to frequent visits of people with residence outside the region, the number of people active in risk areas is much higher than the number of inhabitants used for the relative comparison above. On the other hand, it was shown that in the vast majority of the disease cases, the area of infection coincides with the patient’s residence [37]. The authors hypothesize that the trend in counter-urbanization (change of residence from urban to rural environments) may have an impact on tick-borne disease incidence in the Czech Republic [38].

The non-human associated factors that may influence the difference in the rate of TBE disease case incidence between the two regions will be discussed further. Despite the fact that the number of sampling sites differs in Germany and the Czech Republic, the density of sampling sites still allows the comparison of each area and is comparable to similar studies. The average density of host-seeking ticks was statistically significantly higher in the South Bohemian than in Bavarian study sites. This finding was further supported by the result of the tick density model, predicting higher portions of areas classified as high, very high, and maximum risk for South Bohemia than Bavaria (Table 5). As the risk of vector-borne human disease case occurrence corresponds with the rate of contact between a susceptible person and infected vector [11,39], increased activity of the vector itself increases the risk. This is especially valid in the case of Lyme borreliosis, where the pathogen seems to be present in most of the *I. ricinus* populations. Presence of *B. burgdorferi* s.l. was confirmed for all the sampling plots in our study [18,19]. It was previously noticed that the transmission cycles of TBEV are more fragile and tend to be scattered in smaller geographically-restricted areas [40]. On the other hand, in the case of TBEV, a high tick density may be a crucial factor in natural focus development and stability [41,42]. Tick-borne encephalitis virus positive sites show a higher host-seeking tick density than TBEV negative ones [18,43]. Apart from the higher prevalence of *Borrelia* in adult ticks from Bavaria, no other differences in the prevalence rate of pathogens in ticks were found in the comparison of the two regions. In general, the variability in the density of host-seeking ticks was much higher than the variability in pathogen prevalence. Therefore, in this situation, the risk of infection is rather influenced by the tick abundance compared to the pathogen prevalence. The difference in the incidence of TBE in South Bohemia and Bavaria may then be assigned not only to differences in human behavior, but also to different levels of (infected) tick density, and thus general suitability of the area for *I. ricinus* occurrence.

The main aim of our study was to offer the general public easy access to information on the risk of infection by tick-borne diseases. Infection risk estimation based on disease incidence has several advantages—provides information about epidemiological significance, takes into account insusceptible or immune persons, application of preventive measures, etc. [10,44,45]. On the other hand, such systems are, to a large extent, affected by the rate of human activity, which may result in undervaluation of the real acarological risk in less commonly visited natural areas [44]. Similarly, in regions with an extremely high vaccination rate (like Austria in the case of TBEV), the incidence-based systems are not applicable for disease risk estimation at all [46]. Furthermore, there are other sources of distortion associated with low accuracy in the geographic localization of the infected vector attack, differences in case definitions, differences in the awareness of the medical doctors, and loss of resolution due to legal or other reasons [10,44,45]. Recently, as an alternative approach, surveillance systems based on the estimation of TBE seroprevalence in sentinel animals were proposed [47]. The authors also mention that the detection of TBEV in ticks is not a suitable indicator for TBE infection risk estimation. Our study shows that at least in areas with high TBEV occurrence, this approach brings valuable data for disease risk estimation.

The close relationship between the abundance of vector species and various environmental variables, together with the increased availability of high-resolution sources of geo-referenced environmental data and growing capabilities of GIS tools, have made it possible to construct (infected) vector-based models [44,48]. Such systems allow the prediction of infection risk that is encountered by a susceptible person entering a certain geographical area. In the case of our model, this means prediction of the density of infected ticks. We have proposed a model predicting the density of host-seeking ticks and probability of tick infection by either LB spirochetes or TBEV.

The potential explanatory variables were similar to those in other tick-borne disease risk models [6,13,17]. All the three models share some of the significant predictors: land surface temperature and NDVI (two of them altitude, orientation of the slope), although the specific forms of the variable expression differ (mean, minimum, month of NDVI reading). NDVI and other remote sensing data-derived indices describing the vegetation cover are frequently used predictors in tick (tick-borne disease) ecological and epidemiological studies [12,13,15,49]. Information on vegetation cover contains not only information on climatic and to some extent even microclimatic conditions, but also information on probable tick host species’ richness and abundance. Nevertheless, characteristics of vegetation cover are usually combined with climatic factors to increase the model accuracy [13,50], as it was shown that variation in climatic conditions may influence the relationship between tick abundance and NVDI [50,51]. Furthermore, data on (adult tick) host availability (typically deer abundance) are frequently powerful predictors of tick density [16,52]. Such data, with a sufficient resolution and comparable quality for the whole area, were not available at the time of the study. 

The purpose of this study was to model the tick-borne disease risk and hence the environmental variables were used as technical correlates. Thus, causality of the relationships is not necessarily direct. A positive correlation of density of host-seeking ticks with land surface temperatures and negative correlation with altitude were recorded, as expected [4,5]. In the case of the prediction of probability of infection, we would assume an indirect interaction mediated by host species composition and ratio between the abundance of transmission competent and incompetent hosts influencing the tick abundance and prevalence of the pathogens [7,9].

Different genospecies of the *B. burgdorferi* s.l. complex display different potential to cause Lyme disease in human [53]. In our study, we have used data on the occurrence of *B. burgdorferi* s.l., regardless of the particular genospecies. Nevertheless, it was previously reported for a part of the same set of tick samples, that the majority of LB spirochete positive ticks were infected by unequivocally pathogenic species (*B. burgdorferi* s. s., *B. afzelii*, *B. garinii*, *B. bavariensis*, *B. spielmanii*) [53], namely more than 96% in South Bohemia [18] and 81% in Lower Bavaria and Upper Palatinate [19]. Furthermore, other genospecies of the *B. burgdorferi* complex (*B. bissetti*, *B. valaisiana*, *B. lusitaniae*) may be at least conditionally pathogenic [53,54]. Therefore, we have used the data on *B. burgdorferi* sensu lato presence in ticks for the model construction.

The quality of the prediction of host-seeking tick density by the model was evaluated using McFadden’s pseudo R^2^. This goodness-of-fit index can be understood as an alternative to OLS coefficient of determination (R^2^); however, as stated by [55], its values tend to be considerably lower than R^2^, for example, values of 0.2 to 0.4 represent an excellent fit of the model. Hence, in our case, the value of 0.203 indicates a model with very good fit. Relatively low ROC values reached by the tick infection probability models indicate that both models are suitable for predictions; however, their predicting ability is limited.

The resolution of the model is crucial in its ability to distinguish small areas of high risk in large low risk areas [10], which is a typical pattern in diseases with natural focality, like TBE. The spatial structure of TBE focus consists of a microfocus, relatively small core area, where the virus is present continuously and a wider surrounding area, where the virus is spread by vertebrate hosts, predominantly rodents [56]. Based on the longitudinal monitoring of two foci identified based on an analysis of human TBE disease cases, Dobler et al. [56] estimated the size of a TBE focus to be about 2500 m^2^. The final resolution of the model (250 × 250 m pixel size) is, in principal, capable of the distinction of potential tick-borne disease foci of a small size. Compared to similar studies [3,6,13], we have attempted to consider a rather large area in order to provide meaningful information to the public. Despite the relatively lower number of sampling sites, we have included the majority of relevant *I. ricinus* habitat types to obtain a reliable risk prediction.

An obvious limitation of the model is that the data were collected in a single season. Annual variations in total tick abundance, as well in the seasonal patterns, were reported previously. Nevertheless, the ratios among different study sites seem to be more stable [39,57,58,59,60,61]. Hence, we consider the proposed model of tick abundance without the influence of season as generally valid in the sense of relative risk prediction, despite the annual variations. Accordingly, the graphical representation of the model shows classes of minimum to maximum risk, with tick density being only an indicative value. Some annual variations were also observed in the prevalence of TBEV and LB spirochetes [58,62,63]. Nevertheless, concerning the overall low prevalence of TBEV, the risk is more related to presence/absence of the pathogen, which is geographically stable, at least for shorter periods of time (several years). Concerning the predicted probability of infection by *B. burgdorferi* s.l., it is an image of a single-year situation rather than a prediction valid for several consecutive years.

## 5. Conclusions

To conclude, a considerably higher incidence rate of TBE in South Bohemian parts of the study area compared to Bavarian parts was revealed. These differences should be at least partially assigned to the higher density of host-seeking *I. ricinus* ticks in South Bohemia. Spatial models of tick abundance, probability of tick infection by *B. burgdorferi* and TBEV, and models of the density of infected *I. ricinus* ticks were constructed based on correlations with environmental variables. From our present experience, the final presentation of the data in a form of an interactive online map portal (http://gisak.vsb.cz/klistata/index_en.html) seems to be an ideal way to pass the information to the public.

## Figures and Tables

**Table 1:** Environmental and other accessory data used for model construction and testing *.

Type of Data	Data	Description	Source Czech Rep.	Source Bavaria
Epidemiological	number LB cases	disease cases per municipality	National Institute of Public Health, Prague	RKI, Berlin; local public health authorities
	number TBE cases			
Demographic	number of inhabitants	number of inhabitants per municipality	Czech Statistical Office	Federal Statistical Office Germany
Physical-geographical	altitude	digital elevation model	ArcCR500, ArcDATA, Prague	Vektor 500, ATKIS^®^ Basis DLM, Bayerische Vermessungsverwaltung
	slope			
	exposition			
Climatic	land surface temperature (LST)	MODIS Land Surface Temperature, 1000 m raster	NASA, LP DAAC	NASA, LP DAAC
Vegetation cover	NDVI	MODIS NDVI, 250 m raster	NASA, LP DAAC	NASA, LP DAAC
	forest type	CORINE landcover 2006, 100 m raster	EEA	EEA

* LB—Lyme borreliosis; TBE—tick-borne encephalitis; NDVI—normalized difference vegetation index; RKI—Robert Koch Institute; NASA, LP DAAC—National Aeronautics and Space Administration, Land Processes Distributed Active Archive Center; EEA—European Environment Agency.

**Table 2:** Prevalence of *Borrelia burgdorferi* s.l. in field-collected *Ixodes ricinus* ticks.

Tick Stage	Nymphs		Adults		Total	
	Positive/Tested	Prevalence	Positive/Tested	Prevalence	Positive/Tested	Prevalence
S. Bohemia	1239/9809	12.63%	117/1373	8.52% *	1356/11,182	12.13%
Bavaria	458/3599	12.73%	51/369	13.82% *	509/3968	12.83%
Total	1697/13,408	12.66%	168/1742	9.64%	1865/15,150	12.31%

Statistically significant differences (*p* < 0.05) are indicated by *.

**Table 3:** Minimum infection rate of TBEV in field-collected *Ixodes ricinus* ticks.

Tick Developmental Stage	Nymphs		Adults		Total	
	Positive/Tested	MIR	Positive/Tested	MIR	Positive/Tested	MIR
S. Bohemia	54/18,829	0.29%	10/1228	0.81%	64/20,057	0.32%
Bavaria	20/8203	0.24%	3/602	0.50%	23/8805	0.26%
Total	74/27,032	0.27%	13/1830	0.71%	87/28,862	0.30%

MIR—minimum infection rate. Statistically significant differences (*p* < 0.05) are indicated by *.

**Table 4:** Correlation of potential explanatory variables with the density of host-seeking *Ixodes ricinus* ticks *.

Variable	Spearman R	*p*-Level
LST date of collection	0.136	0.096
**LST seasonal mean**	**0.323**	**0.001**
LST seasonal minimum	−0.174	0.033
LST seasonal maximum	0.231	0.004
**Altitude**	**0.089**	**0.277**
Deciduous forest proportion	−0.073	0.373
Coniferous forest proportion	0.016	0.843
Mixed forest proportion	0.207	0.011
Other veg. cover proportion	−0.174	0.034
Proportion slope over 5%	−0.028	0.732
Proportion northwards	0.053	0.523
**Proportion southwards**	**−0.267**	**0.001**
Average altitude	−0.008	0.924
NDVI Date of collection	−0.055	0.501
NDVI May	−0.047	0.569
NDVI June	−0.090	0.276
NDVI July	−0.226	0.005
**NDVI August**	**−0.257**	**0.002**
NDVI September	−0.235	0.004
NDVI seasonal mean	−0.244	0.003
NDVI July-September mean	−0.286	0.001
NDVI seasonal maximum	−0.126	0.124
NDVI seasonal minimum	−0.310	0.001

* LST—land surface temperature, NDVI—normalized difference vegetation index recorded in the indicated month of the year of tick sampling. Seasonal minimum, maximum, and mean values were calculated for the period May–September. Proportions of area of a particular character and average altitude were calculated from a pixel of sampling site and eight surrounding pixels, pixel size—250 m. Variables included in the initial model are indicated in bold.

**Table 5:** Parameter estimates—host-seeking tick density model *.

Parameter	Coefficient	Standard Error	Wald Statistics	Lower Conf. Limit (95%)	Upper Conf. Limit (95%)	*p*
**Intercept**	5.9739	0.1178	50.7313	5.7429	6.2045	<0.001
**LST Seasonal Mean**	0.0199	0.0039	5.0860	0.0122	0.0276	<0.001
**Altitude**	−0.0008	0.0001	−9.5683	−0.0009	−0.0006	<0.001
**Proportion Southwards**	−0.4925	0.0208	−23.6768	−0.5333	−0.4518	<0.001
**NDVI August**	−1.0467	0.0601	−17.4058	−1.1641	−0.9284	<0.001
**Period—Spring**	0.3935	0.0144	27.2851	0.3652	0.4218	<0.001
**Period—Summer**	0.0968	0.0154	6.2837	0.0666	0.1269	<0.001

* LST—land surface temperature, NDVI August—normalized difference vegetation index recorded in August. Seasonal mean LST values were calculated for the time period of May–September. Proportions of area of a particular character (area of south–facing slopes) were calculated from the total area of the pixel of sampling site and eight surrounding pixels, pixel size—250 m.

**Table 6:** Parameter estimates for the model of probability of *B. burgdorferi* infection of ticks *.

Parameter	Coefficient	Standard Error	Wald Statistics	Lower Conf. Limit 95%	Upper Conf. Limit 95%	*p*
**Intercept**	0.5460	0.2657	2.0553	0.0243	1.0674	0.040
**NDVI May**	−1.0642	0.167	−6.3716	−1.3926	−0.7328	<0.001
**Proportion northwards**	−0.1532	0.0422	−3.6290	−0.2360	−0.0713	<0.001
**Altitude**	0.0005	0.0002	3.0032	0.0002	0.0008	0.003
**LST seasonal mean**	−0.0514	0.0084	−6.159	−0.0679	−0.0351	<0.001

* LST—land surface temperature, NDVI May—normalized difference vegetation index recorded in May. Seasonal mean LST value was calculated for May-September. Proportions of area of a particular character (area of north-facing slopes) were calculated from the total area of the pixel of sampling site and eight surrounding pixels area, pixel size—250 m.

**Table 7:** Parameter estimates for the model of probability of TBEV infection in ticks *.

Parameter	Coefficient	Standard Error	Wald Statistics	Lower Conf. Limit 95%	Upper Conf. Limit 95%	*p*
**Intercept**	−2.2753	0.4913	−4.6309	−3.2658	−1.3322	<0.001
**LST seasonal minimum**	−0.0386	0.0151	−2.5633	−0.0682	−0.0091	0.010
**Proportion mixed forest**	0.2188	0.0858	2.5497	0.0497	0.3884	0.011
**NDVI May**	−2.5262	0.5601	−4.5103	−3.6222	−1.4000	<0.001
**NDVI seasonal maximum**	2.3233	0.6853	3.3903	0.9201	3.6734	0.001

* LST—land surface temperature, NDVI May—normalized difference vegetation index recorded in May. LST seasonal minimum and maximum values were calculated for May-September. Proportions of area of a particular character (area covered by mixed forests) were calculated from the total area of the pixel of sampling site and eight surrounding pixels, pixel size—250 m.

**Table 8:** Comparison of area classification (prediction of tick activity without influence of season) between the region of South Bohemia (Czech Republic) and the regions of Lower Bavaria and Upper Palatinate in Bavaria (Germany).

Category (Ticks/100 m^2^)	South Bohemia		Lower Bavaria and Upper Palatinate	
	Number of Pixels	Portion of the Whole Area	Number of Pixels	Portion of the Whole Area
0–water bodies	2451	1.7%	789	0.5%
0–15 minimum	91 832	63.8%	115 708	66.6%
15–25 low	6741	4.7%	11 626	6.7%
25–35 increased	20 792	14.5%	28 341	16.3%
35–45 high	19 236	13.4%	16 446	9.5%
45–55 very high	2602	1.8%	746	0.4%
more than 55–maximum	212	0.1%	17	0.0%
Total	143 866	100%	173 673	100%
